# Supplementary material for: The nuclease EXO1 promotes genomic instability by degrading nascent DNA in BRCA-proficient cells
Source: Nat Commun. 2026 Feb 25;17:3169. doi: 10.1038/s41467-026-69981-1 (PMC13046728; doi:10.1038/s41467-026-69981-1)
Supplement: Supplementary file 1 — Supplementary Information [file 41467_2026_69981_MOESM1_ESM.pdf]

Supplementary Information

The nuclease EXO1 promotes genomic instability by degrading nascent DNA in BRCA-proficient cells

Nusawardhana, Nicolae, and Moldovan

a

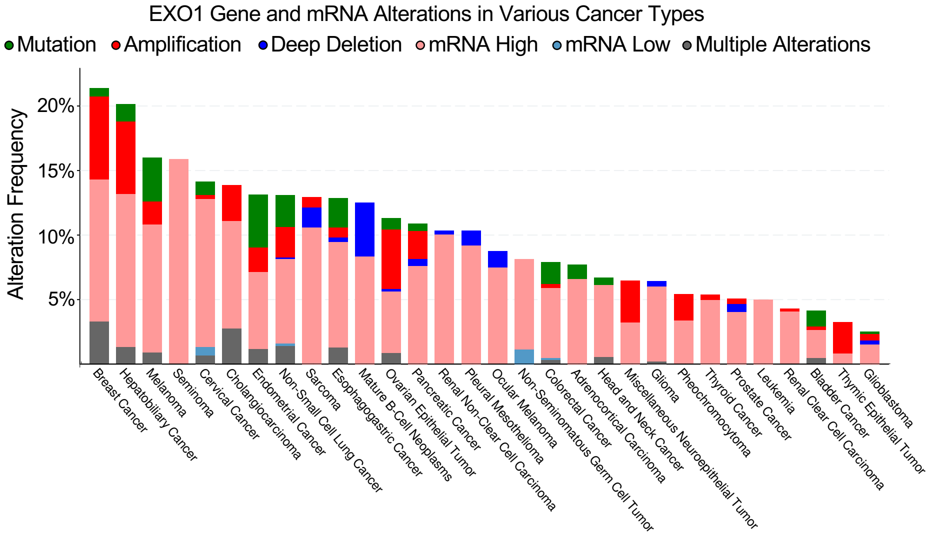

b

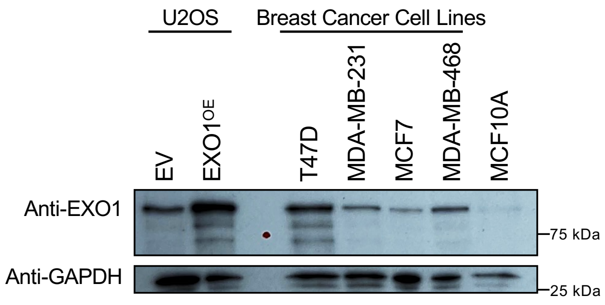

Supplementary Figure 1. EXO1 protein expression in breast tumors.

a. Analysis of EXO1 alterations in TCGA PanCancer Atlas samples show that EXO1 is predominantly overexpressed rather than inactivated. Cancer types are ranked based on the proportion of tumors with EXO1 alterations. All analyses were performed using cBioPortal.org on February 24th 2025. All TCGA PanCancer Studies were selected. Samples were queried by gene name (namely EXO1), with Mutations, Structural Variant, Putative copy number alterations, and mRNA expression selected as Genomic Profiles. The graph shown under Cancer Types Summary is presented.

b. Western blot showing the expression of EXO1 protein in breast cancer cell lines (T47D, MDA-MB-231, MCF7, MDA-MB-468), normal breast epithelial cell line MCF10A, as well as in the U2OS overexpression system we generated.

Source data are provided as a Source Data file.

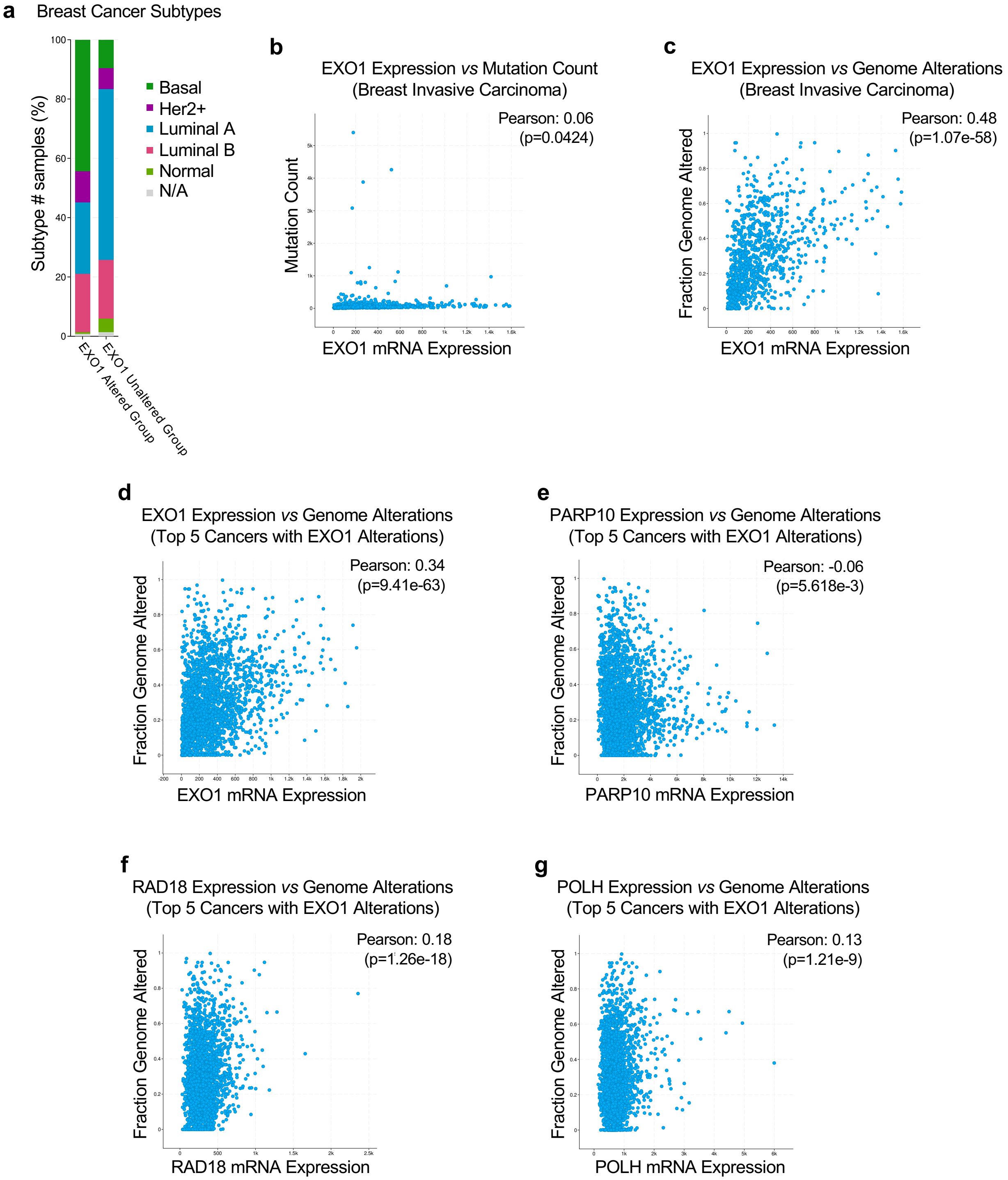

Supplementary Figure 2. EXO1 expression in tumors correlates with genomic instability.

a. Basal-like breast cancer is present at higher rates in breast cancer tumors with EXO1 alterations in the breast invasive carcinoma TCGA PanCancer Atlas dataset. All analyses were

performed using cBioPortal.org on February 24th 2025. The Breast Invasive Carcinoma (TCGA, PanCancer Atlas) dataset was selected. Samples were queried by gene name (namely EXO1), with Mutations, Structural Variant, Putative copy number alterations and mRNA expression selected as Genomic Profiles. The graph shown under Comparison/Survival, Clinical tab is presented.

b,c. Correlation of EXO1 mRNA expression levels with mutations counts (**b**) and genome alteration (**c**) in the breast invasive carcinoma TCGA PanCancer Atlas dataset. All analyses were performed using cBioPortal.org on February 24th 2025. The Breast Invasive Carcinoma (TCGA, PanCancer Atlas) dataset was selected. Samples were queried by gene name (namely EXO1), with Mutations, Structural Variant, Putative copy number alterations and mRNA expression selected as Genomic Profiles. The graphs presented were obtained using the Plots function, using the following axis parameters: Horizontal Axis Data Type: mRNA / mRNA Expression; Vertical Axis Data Type: Clinical Attribute / Mutation Count (**b**) or Clinical attribute/Fraction Genome Altered (**c**).

d-g. Correlation of the mRNA expression levels of EXO1 (**d**), PARP10 (**e**), RAD18 (**f**) and POLH (**g**) with genome alteration in a pooled dataset of breast invasive carcinoma, liver hepatocellular carcinoma, skin cutaneous melanoma, testicular germ cell tumors and cervical squamous cell carcinoma TCGA PanCancer Atlas samples. All analyses were performed using cBioPortal.org on February 24th 2025. The Breast Invasive Carcinoma (TCGA, PanCancer Atlas), Liver Hepatocellular Carcinoma (TCGA, PanCancer Atlas), Skin Cutaneous Melanoma (TCGA, PanCancer Atlas), Testicular Germ Cell Tumors (TCGA, PanCancer Atlas), and Cervical Squamous Cell Carcinoma (TCGA, PanCancer Atlas) datasets were selected. Samples were queried by gene name (namely EXO1), with Mutations, Structural Variant, Putative copy number alterations and mRNA expression selected as Genomic Profiles. The graphs presented were obtained using the Plots function, using the following axis parameters: Horizontal Axis Data

Type: mRNA / mRNA Expression; Vertical Axis Data Type: Clinical attribute/Fraction Genome Altered.

Source data are provided as a Source Data file.

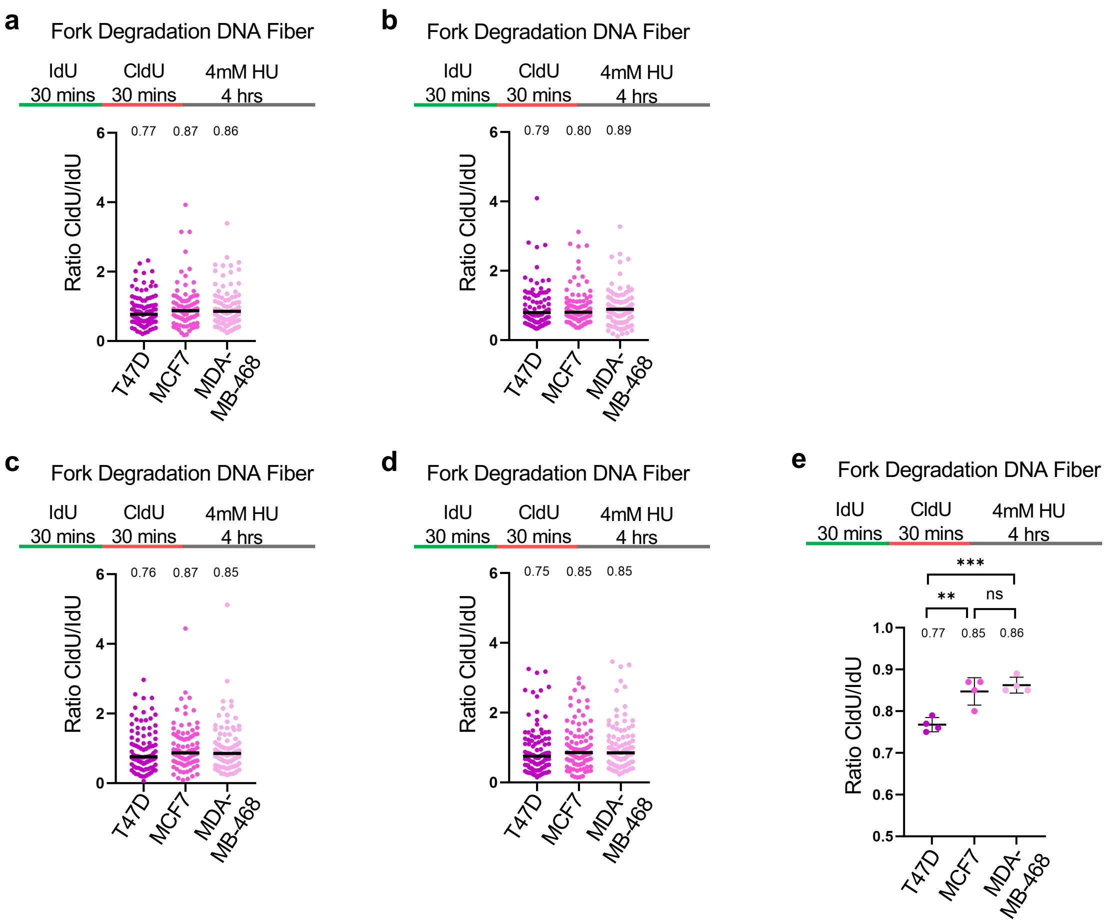

Supplementary Figure 3. Replication fork degradation in breast cancer cell lines.

a-d. DNA fiber combing assays showing fork degradation induced by treatment with 4mM HU for 4hrs in breast cancer cell lines T47D, MCF7 and MDA-MB-468. Four independent experiments are shown. The ratio of CldU to IdU tract lengths is presented, with the median values marked on the graphs and listed at the top. At least 80 tracts were quantified for each sample. Schematic representations of the assay conditions are shown at the top.

e. Quantification of median CldU/IdU ratios from the four independent experiments presented. The mean values and standard deviations are shown. Asterisks indicate statistical significance (t-test, two-tailed, unpaired).

Source data are provided as a Source Data file.

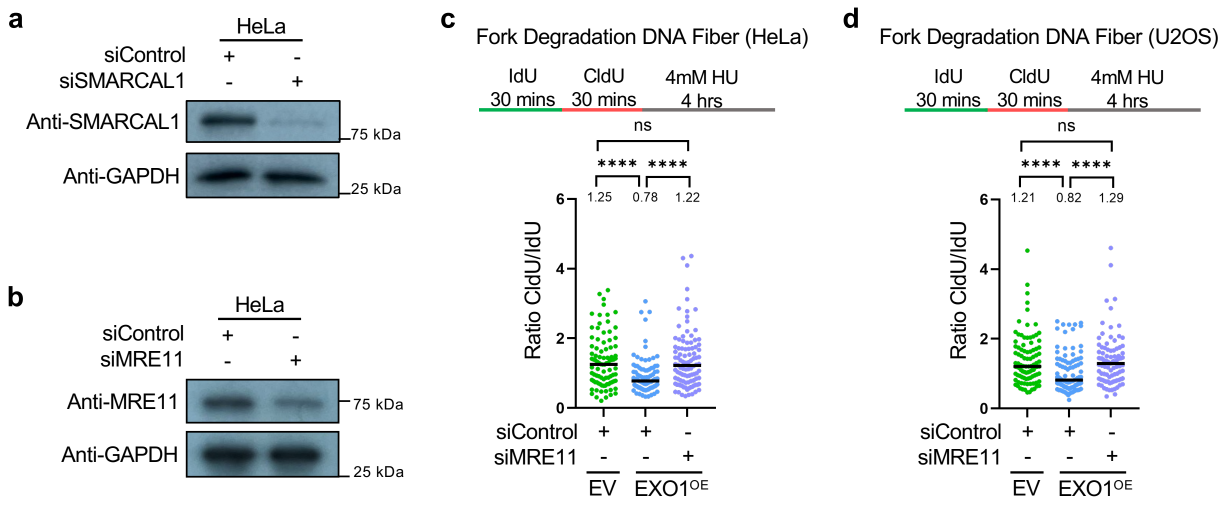

Supplementary Figure 4. Replication fork degradation in breast cancer cell lines.

a,b. Western blots showing the siRNA-mediated knockdown of SMARCAL1 (**a**) and MRE11 (**b**) in HeLa cells.

c,d. DNA fiber combing assay showing that fork degradation induced by treatment with 4mM HU for 4hrs in EXO1-overexpressing HeLa (**c**) and U2OS (**d**) cells is suppressed by knockdown of MRE11. The ratio of CldU to IdU tract lengths is presented, with the median values marked on the graphs and listed at the top. At least 90 tracts were quantified for each sample. Asterisks indicate statistical significance (Mann-Whitney, two-tailed). Schematic representations of the assay conditions are shown at the top.

Source data are provided as a Source Data file.

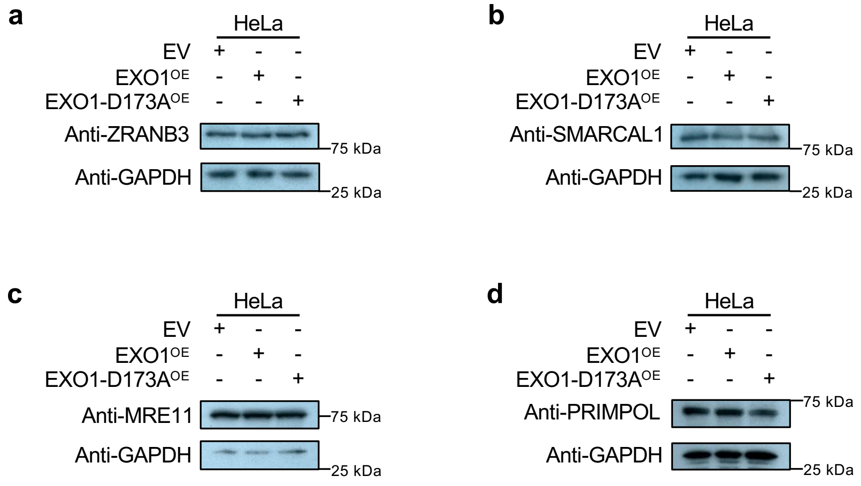

Supplementary Figure 5. EXO1 overexpression does not affect the levels of fork processing factors.

a-d. Western blots showing the protein expression levels of ZRANB3 (**a**), SMARCAL1 (**b**), MRE11 (**c**) and PRIMPOL (**d**) in EXO1-overexpressing HeLa cells.

Source data are provided as a Source Data file.
